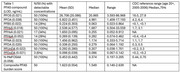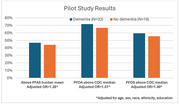# The PFAS VascCog Longitudinal Study: Pilot Data

**DOI:** 10.1002/alz70860_096526

**Published:** 2025-12-23

**Authors:** Hannah Gardener, Bonnie Levin, Kurunthachalam Kannan, Tatjana Rundek

**Affiliations:** ^1^ Department of Neurology, University of Miami Miller School of Medicine, Miami, FL, USA; ^2^ University of Miami Miller School of Medicine, Miami, FL, USA; ^3^ Wadsworth Center, New York State Department of Health, Albany, NY, USA

## Abstract

**Background:**

Per‐ and polyfluoroalkyl substances (PFAS) are persistent organic pollutants found in furniture, cookware, home décor, clothing, firefighting foam, food packaging, and contaminants in food and water. Their ubiquitous exposure, resistance to environmental degradation, and bioaccumulation make PFAS an environmental health priority. Growing evidence of deleterious effects on vascular risk factors and neurotoxicity suggest that PFAS exposure increases dementia risk, but empirical data are limited, weak, and inconsistent. Prospective cohort studies linking PFAS and dementia are lacking. We present pilot data of our new prospective cohort study to measure serum concentrations of 13 PFAS at two time points in relation to dementia in the multi‐ethnic population‐based Northern Manhattan Study (NOMAS).

**Method:**

To establish feasibility of measuring PFAS from frozen serum and public health relevance for our study, we conducted a pilot study of 50 NOMAS participants (32 dementia, 18 without dementia, mean age=80±6, 14% male, 18% White, 12% Black, 68% Hispanic) chosen to be similar by age, sex, and education. A validated method to analyze PFAS in serum incorporates a simplified sample‐preparation protocol based on hybrid‐solid‐phase extraction tailored to measurement by ultra‐performance liquid chromatography coupled to tandem mass spectrometry. A standardized adjudication system classified dementia based on a series of comprehensive neuropsychological assessments, informant questionnaires, and functional ability.

**Result:**

All participants had detectable PFAS, with variability in concentrations, consistent with 2005‐2006 national CDC data (Table). Individual PFAS were combined into a composite PFAS burden score using item response theory, also demonstrating variability. Dementia cases trended towards being more likely to be above the mean for total PFAS burden in the pilot sample, and to have PFOA and PFOS concentrations above the median for the CDC reference sample, *p* >0.05 (Figure).

**Conclusion:**

Results suggest feasibility of the new PFAS VascCog Longitudinal Study, quantifying PFAS concentrations in stored serum, calculating a composite measure of total PFAS burden, consistent with population statistics, supporting public health generalizability. Pilot data support our hypothesis that higher PFAS exposure increases dementia risk. Future findings can provide novel mechanistic insight into the impact of PFAS on cognitive health, identify new avenues for disease modification, and support PFAS regulatory efforts.